# Impact of demographic disparities in social distancing and vaccination on influenza epidemics in urban and rural regions of the United States

**DOI:** 10.1186/s12879-019-3703-2

**Published:** 2019-03-04

**Authors:** Meghendra Singh, Prasenjit Sarkhel, Gloria J. Kang, Achla Marathe, Kevin Boyle, Pamela Murray-Tuite, Kaja M. Abbas, Samarth Swarup

**Affiliations:** 1 Network Dynamics and Simulation Science Laboratory, Biocomplexity Institute of Virginia Tech, Blacksburg, 24060 Virginia USA; 2 Department of Economics, University of Kalyani, Nadia, 741235 West Bengal India; 3 Department of Population Health Sciences, Virginia Tech, Blacksburg, 24060 Virginia USA; 4 Department of Agricultural and Applied Economics, Virginia Tech, Blacksburg, 24060 Virginia USA; 5 Department of Civil Engineering, Clemson University, Clemson, 29634 South Carolina USA; 6 Department of Infectious Disease Epidemiology, London School of Hygiene & Tropical Medicine, London, WC1E7HT UK; 7 Biocomplexity Institute & Initiative, University of Virginia, Charlottesville, 22908 Virginia USA; 8 Department of Public Health Sciences, University of Virginia, Charlottesville, 22908 Virginia USA

**Keywords:** Influenza, Epidemics, Self-protective behaviors, Health disparities

## Abstract

**Background:**

Self-protective behaviors of social distancing and vaccination uptake vary by demographics and affect the transmission dynamics of influenza in the United States. By incorporating the socio-behavioral differences in social distancing and vaccination uptake into mathematical models of influenza transmission dynamics, we can improve our estimates of epidemic outcomes. In this study we analyze the impact of demographic disparities in social distancing and vaccination on influenza epidemics in urban and rural regions of the United States.

**Methods:**

We conducted a survey of a nationally representative sample of US adults to collect data on their self-protective behaviors, including social distancing and vaccination to protect themselves from influenza infection. We incorporated this data in an agent-based model to simulate the transmission dynamics of influenza in the urban region of Miami Dade county in Florida and the rural region of Montgomery county in Virginia.

**Results:**

We compare epidemic scenarios wherein the social distancing and vaccination behaviors are uniform versus non-uniform across different demographic subpopulations. We infer that a uniform compliance of social distancing and vaccination uptake among different demographic subpopulations underestimates the severity of the epidemic in comparison to differentiated compliance among different demographic subpopulations. This result holds for both urban and rural regions.

**Conclusions:**

By taking into account the behavioral differences in social distancing and vaccination uptake among different demographic subpopulations in analysis of influenza epidemics, we provide improved estimates of epidemic outcomes that can assist in improved public health interventions for prevention and control of influenza.

**Electronic supplementary material:**

The online version of this article (10.1186/s12879-019-3703-2) contains supplementary material, which is available to authorized users.

## Background

According to the World Health Organization, influenza outbreaks occur annually and affect 10–20% of the U.S. population and result in about a billion cases of infections globally per year [1]. Two primary self-protective ways to reduce influenza infection include pharmaceutical measures such as vaccination, anti-viral medications, and non-pharmaceutical interventions such as social distancing, hand washing, fluid intake and cough-etiquettes [2, 3]. During an influenza pandemic, when a novel viral strain is encountered for which vaccines are not available, non-pharmaceutical interventions and antivirals are the only viable way to support early mitigation efforts, and indeed existing research has shown their effectiveness in delaying and containing influenza pandemics [4–6].

Mathematical models of influenza transmission often incorporate these protective behaviors to predict the likely outcomes of the disease under different scenarios in order to aid public health decision making. Predictions that do not take behavioral dynamics into account may be unreliable, and moreover, unable to effectively inform public health policies, especially the ones that target individual-level behaviors [7]. In the context of infectious disease modeling, individual level mixing and behavioral heterogeneities are critically important because they significantly affect the transmission pathways of the epidemic [8, 9]. To capture these heterogenities, we use a detailed agent based model in which each individual is endowed with a complete set of demographic and social variables; and the disease propagates on the social contact network. Although this level of detail increases model complexity, it allows for a more realistic representation of the heterogeneity present in the natural system [10]. This model reflects greater epidemic realism by integrating contact network structure, infection dynamics, and detailed individual behavior, which are computationally challenging to implement and remain scarce [11].

Although the use of individual-based models in epidemiology is becoming more common, assignment of various behavioral parameters to individuals is still done uniformly, i.e., behaviors are probabilistically uniformly assigned to individuals in the population to study their impact on the epidemic dynamics. There are studies in the literature that measure the compliance to protective behaviors based on demographics, but they do not capture their effect on the disease spread. For example, studies [3, 12] have identified demographic determinants of protective behaviors but their impacts on controlling the spread of the disease have not been measured. There are many reasons why this gap exists in the literature. In order to carry out such an analysis, one needs (a) survey data to assess the actual level of compliance based on demographics; (b) a detailed model in which agents can be assigned unique demographics and behavioral attributes; and (c) a contact network to study the population level effects of these heterogeneous behaviors on epidemic outcomes.

In this research we use survey data to build a model that ties protective health behaviors to the demographics of the individuals [13]. This model helps calculate the probability of compliance to each health behavior, for each individual given his/her demographic attributes. This is further used to accurately represent behavioral assignments in the population, and then to study their impact on the dynamics of the epidemic. In order to determine the effect of demographic-based behavioral compliance assignment on epidemic outcomes, we simulate an influenza epidemic and compare the results in the two scenarios, (a) individuals follow protective behaviors as predicted by their demographics, referred to as “with-predictors” scenario and (b) individuals follow protective behaviors based on the distribution of protective behaviors in the survey responses, independent of their demographics, referred to as “without-predictors” scenario. The results show that epidemic outcomes based on (a) are significantly worse than those based on (b).

## Methods

### Model to estimate compliance probabilities of preventive behaviors

A nationwide survey of 2168 respondents, conducted by the Gfk Group (Gfk.com) in 2016, recorded demographics and preventive health behaviors in response to a hypothetical influenza-like-illness outbreak. The target population was adults aged 18 and above. It recorded a variety of preventive behaviors such as vaccine uptake, social distancing, adoption of personal hygiene such as washing hands, wearing masks, covering cough etc. Our focus here is on two types of preventive behaviors, i.e., vaccination and social distancing. The individuals may adopt any one of them or both or none.

We model the choice of selecting a preventive behavior as a multinomial logit (MNL) model. This is a standard modeling framework when individuals face multiple choices that are not ordered. The response variable *y*_*nj*_ is defined as the observed choice of behavior *j*: vaccination, social distancing, both vaccination and social distancing or neither adopted by the n^th^ individual. In order to make the choice set exhaustive we also include the option that the individual might not adopt any of the three alternatives [14, 15]. Thus the preventive behaviors are indexed as *j*=1,2,3,4 such that *y*_*n*1_ indicates that individual *n* chooses vaccination, *y*_*n*2_ indicates choice of social distancing, *y*_*n*3_ indicates both and *y*_*n*4_ indicates neither. Thus, unordered condition applies because these are mutually exclusive choices and no assumption is imposed regarding households’ ranking of the alternatives.

We further assume that person *n*’s utility function for the four protection alternatives is given by *U*_*nj*_=*V*_*nj*_+*ε*_*nj*_, where *V*_*nj*_ is the deterministic part of the utility function (often called representative utility) and $V_{{nj}}=X_{n}^{\prime }\beta _{j}$. Here, *X*_*n*_ denotes the respondent’s characteristics like age, gender and income. The joint density of the random error vector can be denoted as *f*(*ε*) and used to assess the probability of the choice of different behaviors. Person *n* chooses behavior *i* if it provides higher utility than the three other behavior. The probability of choosing the precautionary behavior *i*, where *i* is one of the four choice alternatives, can be written as [16]:

$$P(y_{{ni}}) = \begin{array}{ll} P(U_{{ni}} > U_{{nj}}) & \forall i \neq j \\ P(\epsilon_{{nj}} - \epsilon_{{ni}} < V_{{ni}} - V_{{nj}}) & \forall i \neq j \end{array} $$

Using *f*(*ε*) this cumulative probability can be rewritten as [15]

1 $$ P(y_{{ni}}) = \int_{\epsilon} I(\epsilon_{{nj}} - \epsilon_{{ni}} < V_{{ni}} - V_{{nj}},\ \forall i \neq j) f(\epsilon) d\epsilon  $$

Where *I*(.) is an indicator function that equals 1 if the term in the parentheses is true, and is 0 otherwise. Different discrete choice models can be obtained from assigning different specifications for *f*(*ε*). The multinomial logit model is obtained by assuming that each *ε*_*nj*_ is independent, identically distributed (iid) extreme value that is also called Gumbel or Type-1 value distribution. Thus, $\phantom {\dot {i}\!}f(\epsilon _{{nj}}) = e^{-\epsilon _{{nj}}} e^{-e^{-\epsilon _{{nj}}}}$ and the cumulative distribution is $F(\epsilon _{{nj}}) = e^{-e^{-\epsilon _{{nj}}}}$. This distributional assumption entails a closed form solution for the multidimensional integral [15]. If two random error terms are iid extreme value distributed, their difference follows a logistic distribution.

The probability of choosing the precautionary behavior *i* can be written as [16, 17]

2 $$  P_{{ni}} = \frac{e^{V_{{ni}}}}{\sum_{j}{e^{V_{{nj}}}}} = \frac{e^{X^{\prime}_{n} \beta_{i}}}{\sum_{j}{e^{X^{\prime}_{n} \beta_{j}}}}  $$

The coefficients are estimated using maximum likelihood and putting them in Eq. 2 we can predict the probability of the observed behavior. In order to form the likelihood function we assign binary codes to indicate the group membership of the observation [17]. In our case we have four options so we create four binary variables *y*_1_,*y*_2_,*y*_3_,*y*_4_. Thus, if the respondents choose vaccination, i.e., *y*_*n*1_ then *y*_1_=1, and *y*_2_=*y*_3_=*y*_4_=0. If we denote the conditional probability in 2 as *π*_*j*_(*X*) then the likelihood function can be written as

3 $$  l(\beta) = \Pi_{n}\left[\pi_{1}(X_{n})^{y_{1n}} \pi_{2}(X_{n})^{y_{2n}} \pi_{3}(X_{n})^{y_{3n}} \pi_{4}(X_{n})^{y_{4n}}\right]  $$

$\hat {\beta }$ is the maximum likelihood estimator that can be used to predict the probability of preventive behavior. To assess the magnitude of change in probabilities due to a unit change in an explanatory variable, the marginal effect is calculated. The marginal effect of demographic *x*_*k*_ on behavior *j* is measured as

4 $$  \frac{\partial_{p_{j}}}{\partial_{x_{k}}} = p_{j}\left(\beta_{{jk}} - \sum{p_{j}\beta_{{jk}}}\right)  $$

### Synthetic models of two US regions

We use an agent based model to construct synthetic representations of two regions, an urban region, i.e., Miami Dade county in Florida, and a rural region, i.e., Montgomery county in Southwest Virginia. The synthetic populations and the social contact networks of these regions have been developed using a “first principles” approach. The synthetic population is a set of synthetic people and households, located geographically, each endowed with the demographic variables recorded in the US census. A synthetic population integrates a variety of databases from commercial and public sources into a common architecture for data exchange to create realistic attributes of the synthetic individuals. The population synthesis process preserves the confidentiality of the individuals in the original data sets. Joint demographic distributions are reconstructed from the marginal distributions available in typical census data using an iterative proportional fitting (IPF) technique [18–20]. Each household is located geographically using land-use data and data pertaining to transportation networks. The process guarantees that a census of our synthetic population is statistically indistinguishable from the original census.

Next each synthetic person in a household is assigned a set of activities to perform during the day, along with the times when the activities begin and end, as given by an activity survey or time-use survey data. Then an appropriate real location is chosen for each activity for every synthetic person based on a gravity model and data sources such as land use patterns and commercial location data from Dun and Bradstreet. Finally a social contact network is generated in which each synthetic person is deemed to have made contact with a subset of other synthetic people simultaneously present at a location [21–23].

The resulting model is a dynamic representation of human mobility and interaction over the course of a normative day. The induced social contact network is an interaction based graph whose vertices are synthetic people, labeled by their demographics, and edges represent estimated contacts, labeled by duration of contact and type of activity. This social contact network is specific to a geographic location because of its dependence on “contingent realities” for the area – demographics of people who live there and the distribution of actual activity locations. It provides a plausible, bottom-up mechanism for generating large scale social structure without making assumptions about hierarchies [6, 24–26]. The distribution of age and household income in the two model populations used in this study are available in the Additional file 1.

It is important to note that the procedures followed while creating the agent based model provide some theoretical guarantees. For example, the generated synthetic population is guaranteed to match the marginal distributions of the true population. Additionally, the model has been validated in multiple ways. First, it has been shown that the distribution of variables not included in the IPF step (e.g., the number of workers in a household) closely match between the synthetic and true population [27]. Second, it has been shown that the activity profiles of our generated synthetic population better match the true population than previous techniques [28]. Third, the mobility patterns in the synthetic population have been validated using various measures of traffic (e.g., trip counts between zones [21]). Lastly, multiple network structural measures (e.g., distribution of numbers of contacts per person outside of home) obtained from the generated synthetic population show expected patterns (e.g., Power-law distributions), reported in literature [29].

### Interventions

We consider two types of behavioral interventions, vaccination and social distancing. Individuals who are vaccinated become immune to the disease with a probability given by the efficacy of the vaccine. We consider three levels of vaccine efficacy, i.e., 20%, 40% and 60%. For example, if vaccine efficacy is 20%, and the individual takes the vaccine, she will have a 20% chance of becoming immune to influenza.

To simulate social distancing, appropriate edges are removed from the social contact network. Each agent in the network can perform six types of activities, i.e., home, work, school, college, shop and other. The category “other” represents all activities not covered by the first five categories, and are labeled “non-essential” activities. These include social, cultural and sport activities that could be avoided if the person is following self-protective behaviors. In our model when a person is trying to avoid getting infected through social-distancing, all her non-essential activities are stopped. Hence, we remove all social contact network edges that are labeled “other”, for individuals who are deemed compliant to social-distancing.

### Disease model

**Fig. 1:**
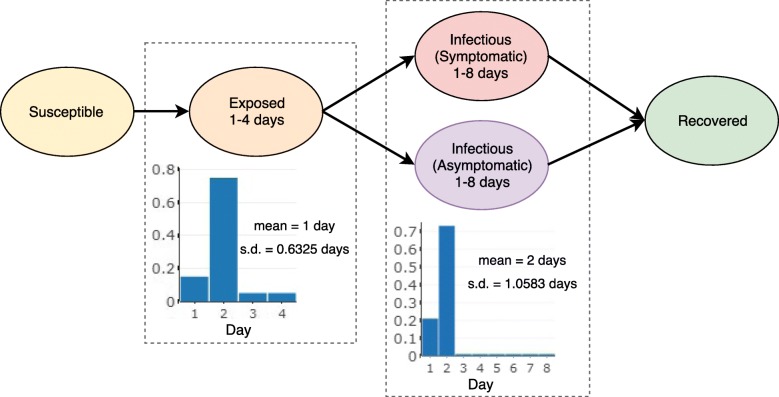
Susceptible Exposed Infectious Recovered (SEIR) disease model used in our simulations to capture the health states of the individuals. Everyone starts out in susceptible state and if infected, moves to the exposed state, followed by one of symptomatic or asymptomatic infectious states, followed by the recovered state. The duration of time spent by an infected individual in the exposed and infectious states is shown in the distributions associated with them

We use Episimdemics, an interaction-based high performance computing simulator for studying epidemic dynamics [30]. A simple 4-state Probabilistic Timed Transition Systems (PTTS) disease model designed for agent-based simulations is used. The four states, Susceptible, Exposed, Infected, and Recovered, depict the change in a susceptible individual’s health status upon getting infected with influenza. These states are also consistent with the SEIR model used in epidemiology. Each agent remains in the susceptible state until it comes into contact with an infected agent through one of its contacts in the social contact network [6, 20]. Figure 1 shows a schematic of the disease model. Upon contact with an infected agent, a susceptible agent *i* transitions to the exposed state with a probability *p*_*i*_ which is computed as:

5 $$ p_{i} = 1 - \exp \left(\tau \sum_{r \in R} N_{r} \ln (1-r s_{i} \rho) \right)   $$

Here, *τ* is the duration of exposure, *R* is the set of infectivities (*r*s) of all the infected agents, *N*_*r*_, co-located with the susceptible agent *i*, *s*_*i*_ is the susceptibility of *i* and *ρ* is the transmission rate, i.e. the probability of a susceptible agent getting infected by an infectious agent per minute of contact time. For a completely susceptible individual *i* (having susceptibility *s*_*i*_=1.0), coming in contact with one completely infectious individual (having infectivity *r*=1.0) for unit exposure time (i.e., *τ*=1.0), the probability of transitioning to the exposed state becomes equal to the disease transmission rate (i.e., *p*_*i*_=*ρ*). The presence of multiple infectious individuals (i.e., *N*_*r*_>1) and/or larger exposure time (i.e., *τ*>1.0) would increase the probability of the susceptible individual getting infected (i.e., *p*_*i*_). Thus, Eq. (5) accounts for this using the summation term in the exponent. When a person becomes infectious, s/he may be asymptomatically infectious or symptomatically infectious. An asymptomatic person is less likely to transmit the disease to susceptible people than a symptomatic person. Initially, everyone in the population is assumed to be susceptible, except for the few individuals with whom the epidemic is seeded. The specific values of disease and simulation parameters used in our simulation experiments are provided in Table 1.

**Table 1:** Simulation parameters, their values and sources, used in the experiments

Variables	Values	Source
Population size (number of individuals)	Miami-Dade (2,169,349), Montgomery (77, 820)	Synthetic populations [5, 24, 41]
Total number of daily contacts between individuals	Miami-Dade (55,187,587), Montgomery (2,019,222)	Synthetic populations [5, 24, 41]
Transmission rate	Miami-Dade (0.00010), Montgomery (0.00018)	Calibrated for each region to generate an attack rate of 25% in both the regions
Attack rate (Cumulative infections)	25%	[42]
Serial interval	Miami-Dade (2.65 days), Montgomery (2.59 days)	Estimated from our disease model [43]
Interventions	Vaccination and/or social distancing	
Latent period	1 day [s.d.: 0.63]	[44, 45]
Infectious period	2 days [s.d.: 1.06]	[44, 45]
Symptomatic proportion	67%	[46]
Asymptomatic infectivity	33%	[47–49]
Vaccine efficacy	20%, 40%, 60%	[50]
Simulation days	200 days	Assumed
Initial number of infections (seeds)	20 for both Miami-Dade and Montgomery	Assumed
Initial number of susceptible individuals	Everyone, except the seeds	Assumed
Number of replicates	25	Assumed
Number of scenarios	2 (compliance probability estimated with, and without predictors)	

### Experiments

We consider two protective behavior assignment scenarios. In the first scenario, we assign the average probability of adapting protective behaviors to agents, consistent with the behavior distribution observed in the survey irrespective of demographics. In this case, behavior assignment is done in such a way that the proportion of individuals who adopt a particular behavior in the simulation is equal to the proportion of survey respondents who report adopting that behavior. In other words, the behavior is averaged across the population; no information about the demographics of an individual is considered while assigning it a compliance behavior.

In the second scenario, we use the multinomial logit model to calculate compliance probabilities as determined by the survey respondents’ demographics. Note that the survey only collects data for individuals who are older than 18 years. Therefore for individuals below 18 years of age in our simulations, we assume that they behave the same as their respective family members and hence we assign them the mean compliance probabilities of their older family members’, as a proxy. Their compliance rates are provided in the Additional file 1. Additionally, for any scenario that considers vaccination, we simulate three levels of vaccine efficacy. Each run is simulated for 200 days and all results are reported as the average of the 25 replicates. A rural and an urban region is used for testing the robustness of the results. The epidemic outcomes are measured by the number of infections when the epidemic peaks (i.e., peak infections), the day of peak infections (i.e., peak day, also known as time-to-peak), and the cumulative number of infections over the simulation duration (i.e., the size of the epidemic). Low values of peak infections and cumulative infections are desirable but high values of peak day are desirable. A baseline case of an unmitigated epidemic that is absent of any protective behavior is also simulated to measure the effectiveness of intervention strategies.

## Results

### Relationship between demographics and behavior adoption

Table 2 shows the results of a multinomial logit (MNL) model which predicts protective behavioral choices as a function of demographics. It shows the MNL model’s coefficient estimates corresponding to the explanatory variables, for each of the three response variables. Here, the explanatory variables are demographics of the surveyed individuals, which are age, gender, family size, family income, and whether the person’s family has children. The three response variables considered are (i) if the individual applied social distancing or not (i.e., Social Distancing), (ii) if the individual took the vaccine or not (i.e., Vaccination), and (iii) if the individual applied both the interventions (i.e., Vaccination & Social Distancing). The base response is that no intervention was applied. To check for the independence of irrelevant alternatives (IIA), we conducted the Hausman test [31, 32]. We failed to reject the hypothesis that IIA holds for the full set of alternatives at less than 1% level of significance.

**Table 2:** Coefficient estimates corresponding to the explanatory variables, for each of the three response variables in the Multinomial Logit regression model

Independent variable	Response variable		
	Social distancing	Vaccination	Vaccination & social distancing
Age of the respondent	0.00655 (0.0252)	-0.0605*** (0.0214)	-0.0576*** (0.0222)
Age squared	8.49*e*^−05^ (0.000263)	-0.000983*** (0.000219)	0.000941*** (0.000226)
Gender	-0.0710 (0.132)	-0.168 (0.115)	-0.159 (0.122)
Household size	0.0905 (0.0674)	0.00538 (0.0624)	0.0317 (0.0650)
Children at home	-0.0341* (0.198)	0.0254 (0.176)	-0.0718 (0.187)
Household income	−3.94*e*^−06^*** (1.48*e*^−06^)	6.31*e*^−06^*** (1.23*e*^−06^)	−2.23*e*−^06^*** (1.36*e*^−06^)
Constant	-1.072* (0.621)	-0.254 (0.543)	0.129 (0.568)
Predicted probabilities	0.16	0.27	0.22
Num. of observations: 2121; Log-likelihood: -2741; Chi-square: 229.1			

The three response variables are: social distancing, vaccination, both vaccination & social distancing. The values in the parentheses are standard errors for the estimates. Statistical significance is shown by: ***, **, and *, which correspond to *p*<0.01, *p*<0.05 and *p*<0.1 respectively

Results in Table 2 show that age follows a non-linear relationship with the response variables as shown by the “square of age” variable which is significant in predicting protective behaviors. This has also been observed by other researchers in the literature [3, 33, 34]. Additionally variables such as gender, household size and the presence of children in the household do not appear to be significant predictors [33, 35]. Among the explanatory variables considered in the regression, age of the respondent and the household income are the most statistically significant predictors. Hence in our simulations, we use age and household income as predictors for generating the probabilities of adopting preventive behaviors. For the scenario that uses demographics as a predictor of compliance to behaviors, we apply the MNL model based probabilities to reflect the level of compliance by each synthetic individual in the simulation.

Note that the estimated coefficients of the MNL model only provide the direction of the change with respect to the base outcome but not the magnitude. To assess the impact of each independent variable on the response, we calculate the marginal effects of the MNL model as shown in Eq. 4.

### Behavioral interventions under different scenarios

We describe results from our experimental scenarios here.

#### (a) No intervention case

To set up a baseline we run an influenza epidemic with no interventions. In this base case, the epidemic infects 25% of the population over its course. The peak of the epidemic occurs close to the 60^*th*^ day in the Montgomery county and 45^*th*^ day in Dade county. Approximately 0.65% of the population is infected in Montgomery and 0.8% is infected in Miami Dade on the peak day.

#### (b) Assigning behaviors independent of demographic predictors

We use survey results to estimate the probability of preventive behavior adoption by setting it equal to the proportion of survey respondents who selected that behavior. Based on these proportions we assign the preventive behaviors to the synthetic individuals in the simulation. We set the probability of adopting vaccination or *P*(*vaccination*) to be 0.264, probability of adopting social distancing or *P*(*socialdistancing*) to be 0.158, probability of adopting both the behaviors or *P*(*both*) to be 0.216 and probability of not adopting any behavior or *P*(*none*) to be 0.361. In this scenario all individuals encounter the same probabilities for behavior assignment, i.e., no demographic information of individuals is used in this assignment.

#### (c) Assigning behaviors based on demographics

**Fig. 2:**
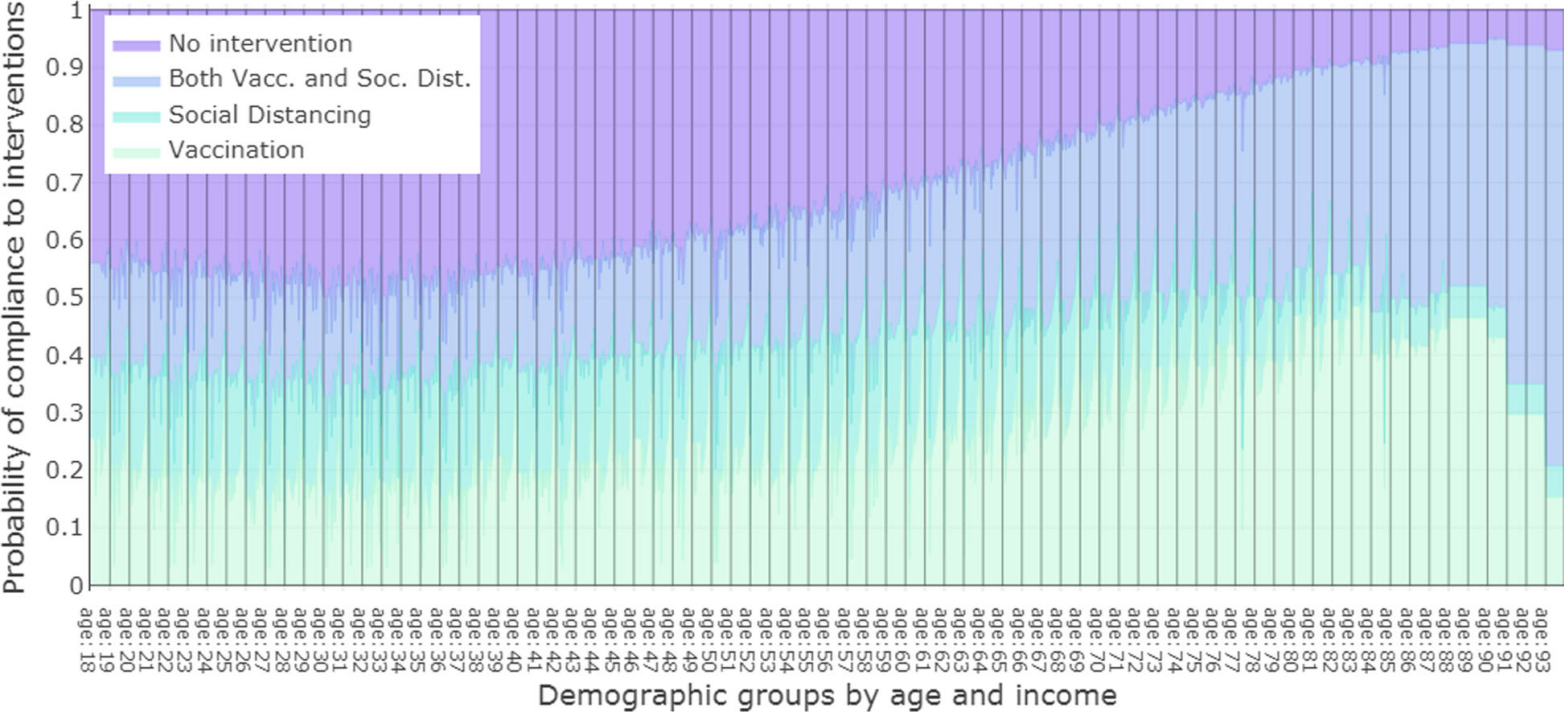
Compliance probability distributions determined by the Multinomial logit model, for the three protective behaviors (vaccination, social distancing, both vaccination and social distancing) across age and income groups present in the survey. The x-axis shows age and income group combinations. Ages are marked on the axis and the interval between two successive age marks is divided into 19 income groups (from less than 5000 USD to greater than 175,000 USD) belonging to that age. The income groups are not marked for the lack of space. The y-axis shows the compliance probability in a stacked bar format where the length of a colored bar represents the compliance probability for the intervention represented by the corresponding color. The plot shows that the overall compliance towards preventive behaviors increases with age

Next we assign the probability of adopting preventive behaviors using the demographics as determined by the MNL model in the Methods section. Based on the regression model results, we find each person’s unique probability based on her age and income category and use it to determine compliance to preventive behaviors during the epidemic. Figure 2 shows the probability of compliance to the three protective behaviors (social distancing, vaccination, both social distancing and vaccination) or none of these three (no intervention) based on age and income. The income categories are not labeled on the x-axis due to lack of space but are reflected in the figure. The compliance to preventive behavior goes up by age, implying older individuals are more compliant.

### Comparison of epidemic outcomes

**Fig. 3:**
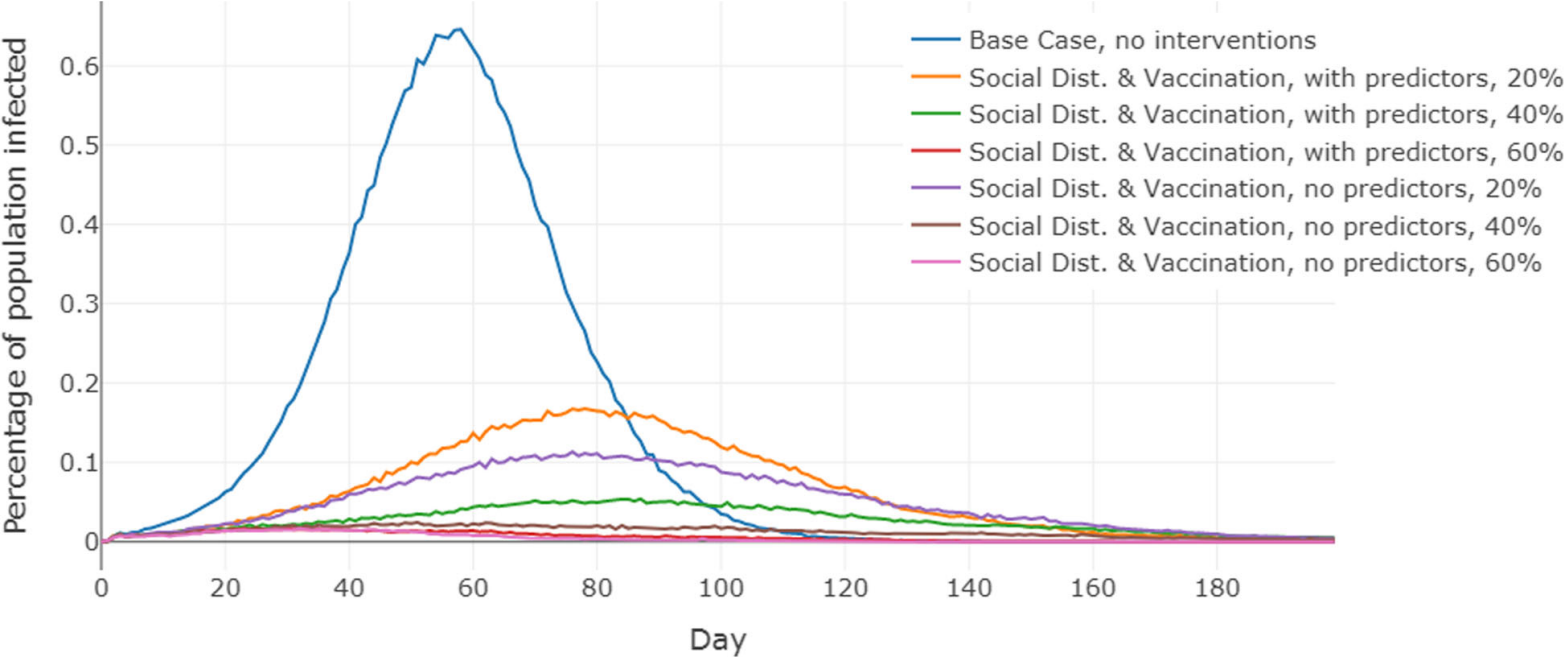
Epidemic curves for the three scenarios in Montgomery county, Virginia. Here, the vertical axis shows the prevalence of Influenza in the population. “Base Case, no interventions” refers to the baseline scenario where we simulate the influenza epidemic without any interventions. “With predictors” refers to the scenario where compliance levels are measured by the MNL model and “no predictors” refers to the scenario where compliance is not determined by demographics, and only average level of compliance is applied. For the last two scenarios, three levels of vaccine efficacy (i.e., 20%, 40% and 60%) were considered

**Fig. 4:**
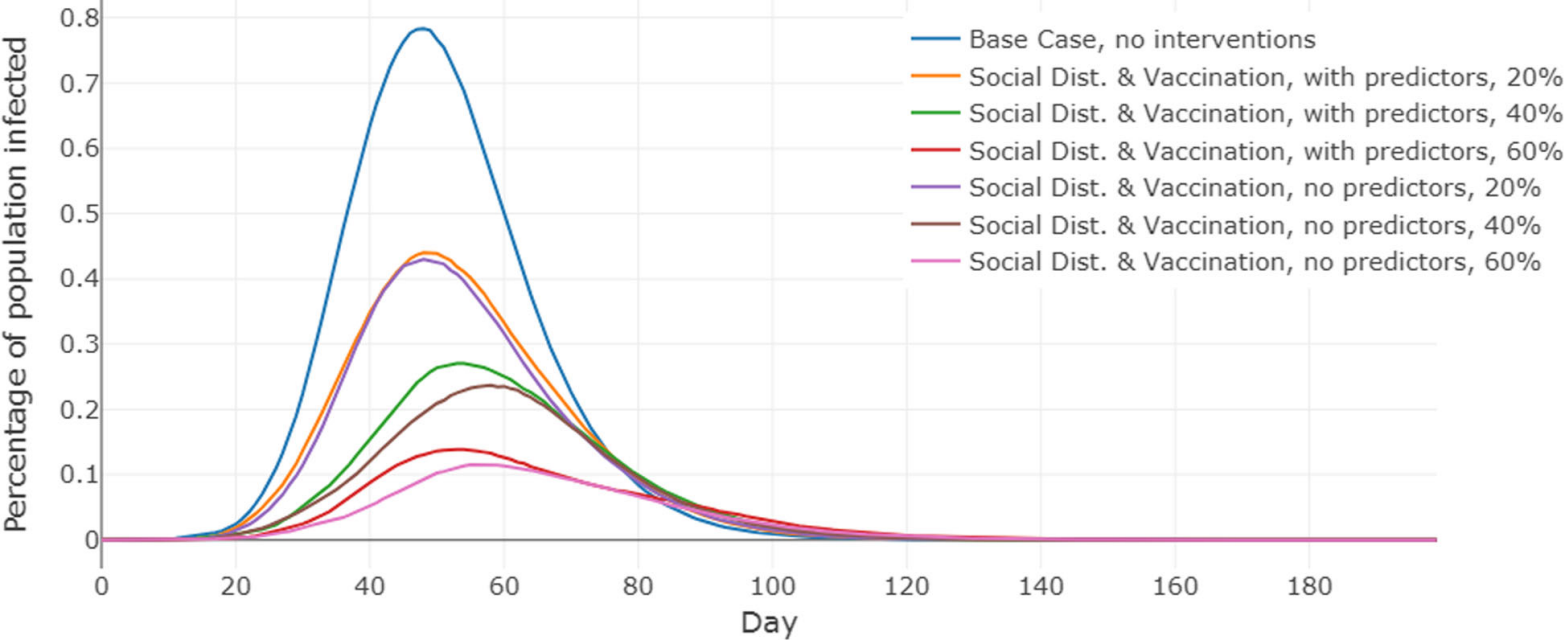
Epidemic curves for the three scenarios in Miami Dade county, Florida. Here, the vertical axis shows the prevalence of Influenza in the population. “Base Case, no interventions” refers to the baseline scenario where we simulate the influenza epidemic without any interventions. “With predictors” refers to the scenario where compliance levels are measured by the MNL model and “no predictors” refers to the scenario where compliance is not determined by demographics, and only average level of compliance is applied. For the last two scenarios three levels of vaccine efficacy (i.e., 20%, 40% and 60%) were considered

The results show that the outcomes of the epidemic are significantly worse when demographics based behavior adaptation probabilities are used, as compared to the case when no demographic predictors are used to determine behaviors. The size of the epidemic is bigger, and the number of infections on the peak day are larger. This holds true for both regions and at almost all levels of vaccine efficacy. Additionally, the differences between the outcomes grow larger as the vaccine efficacy increases. The detailed results for each scenario (reported as the mean of 25 simulation replicates) are presented in Table 3. Figures 3 and 4 show the epidemic curves for the each scenario, including the baseline “no interventions case”.

**Table 3:** Mean epidemic outcomes of 25 replicates for the “With predictor” and “Without predictor” scenarios in the Montgomery and Miami regions, under three vaccine efficacy levels of 20%, 40% and 60%

Regions	Epidemic measure	Scenario	Vaccine efficacy		
			20%	40%	60%
Montgomery, VA	Peak Infections (% of total population)	With predictors	0.2	0.08	0.036
		Without predictors	0.16	0.05	0.033
	**% increase With vs. Without predictors**		**25**	**60**	**9.09**
	Peak day	With predictors	81	83	40
		Without predictors	83	76	37
	**% increase With vs. Without predictors**		**-2.41**	**9.21**	**8.10**
	Total infections (% of total population)	With predictors	11.57	4.28	1.23
		Without predictors	10.07	2.6	0.95
	**% increase With vs. Without predictors**		**14.89**	**64.61**	**29.47**
Miami, FL	Peak infections (% of total population)	With predictors	0.52	0.33	0.18
		Without predictors	0.5	0.29	0.153
	**% increase With vs. Without predictors**		**4**	**13.79**	**18.3**
	Peak day	With predictors	50	54	58
		Without predictors	50	56	62
	**% increase With vs. Without predictors**		**0**	**-3.57**	**-6.45**
	Total infections (% of total population)	With predictors	16.2	10.86	6.48
		Without predictors	15.42	9.64	5.37
	**% increase With vs. Without predictors**		**5.05**	**12.65**	**20.67**

“With predictor” refers to the case where compliance probabilities are derived from the Multinomial Logit (MNL) model and are based on survey participant demographics, whereas “Without predictor” refers to the case where compliance probabilities are equal to mean compliance levels of all participants in the survey, irrespective of their demographics. The highlighted rows show the differences in epidemic outcomes under the two scenarios

To assess the significance-level of the differences between the mean epidemic measures for with and without predictor scenarios in Table 3, we performed the t-test. The results are reported in Table 4. We observe that the difference in the values for peak and total infections obtained with and without demographic predictors is always significant except in Montgomery when vaccine efficacy is 60%. The reason for the low significance in Montgomery is that at 60% vaccine efficacy, the intervention is fairly strong for this rural region and the epidemic almost dies out in both the scenarios as can be seen in Fig. 3. The day on which the peak infections occur do not change significantly between scenarios.

**Table 4:** T-test results for the three epidemic outcomes, with versus without predictors scenarios, in the Montgomery and Miami regions

Geography	Epidemic measure	Scenario	Vaccine efficacy		
			20%	40%	60%
Montgomery, VA	Peak Infections with vs. without predictors	t-statistic	4.27	4.85	1.26
		*p*-value	**9** **.** **1** **6** *e* ^−5^	**1** **.** **3** **3** *e* ^−5^	0.211
	Peak Day with vs. without predictors	t-statistic	-0.31	0.5643	0.6094
		*p*-value	0.755	0.575	0.545
	Total Infections with vs. without predictors	t-statistic	4.53	6.13	2.19
		*p*-value	**3** **.** **9** **4** *e* ^−5^	**1** **.** **5** **9** *e* ^−5^	**0.033**
Miami, FL	Peak infections with vs. without predictors	t-statistic	1.847	7.33	6.32
		*p*-value	**0.0708**	**2** **.** **2** **3** *e* ^−9^	**7** **.** **8** **4** *e* ^−8^
	Peak Day with vs. without predictors	t-statistic	-0.1222	-0.6071	-0.9177
		*p*-value	0.9032	0.5466	0.3633
	Total Infections with vs. without predictors	t-statistic	18.46	35.5	39.06
		*p*-value	**1** **.** **9** **1** *e* ^−23^	**4** **.** **1** **3** *e* ^−36^	**4** **.** **8** **4** *e* ^−38^

The highlighted numbers show statistically significant t-statistic values corresponding to differences in epidemic outcomes

**Fig. 5:**
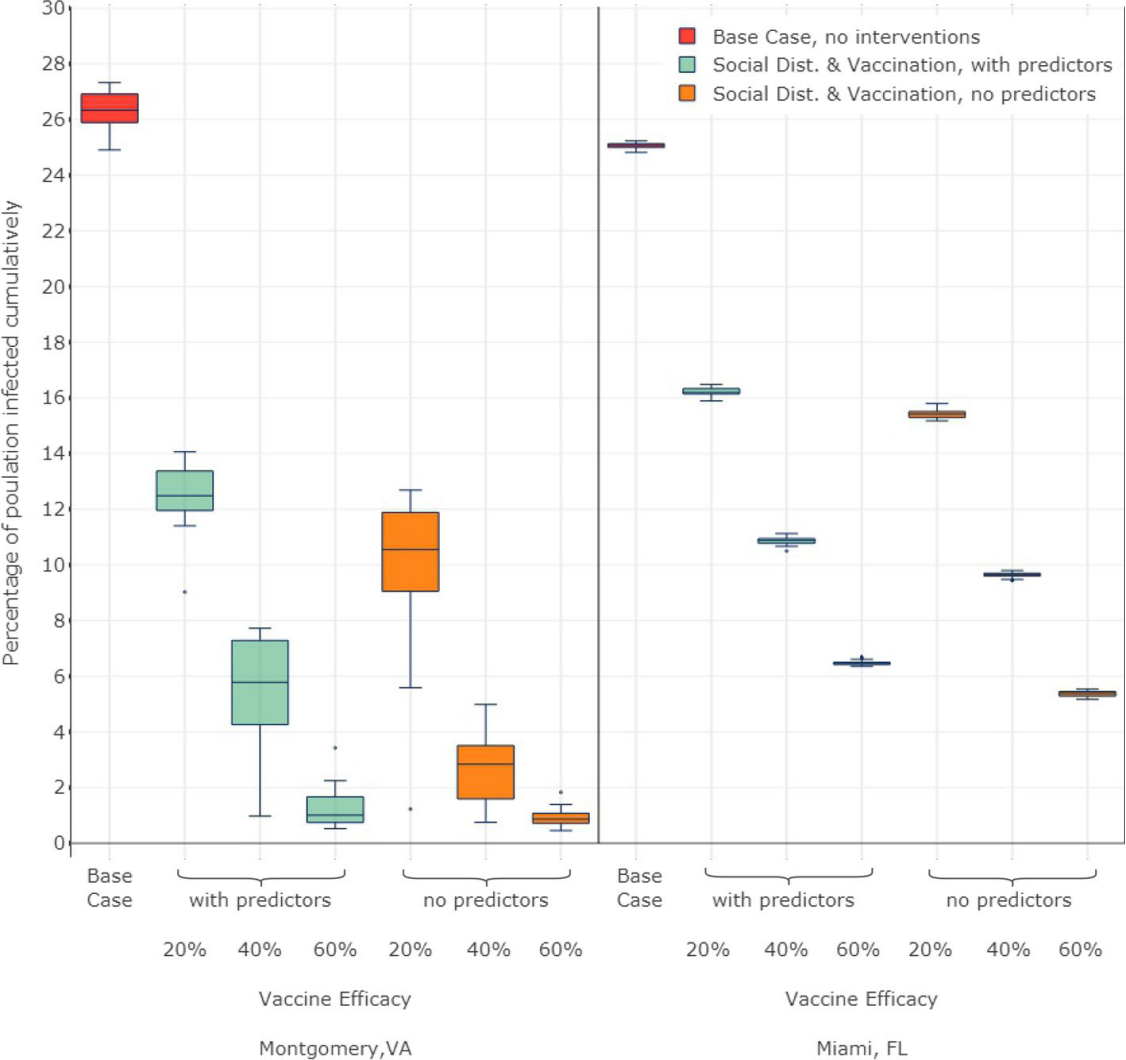
Comparative evaluation of cumulative infections observed for three experimental scenarios (i.e., “base case” in which no preventive behavior is modeled, “With predictors” case in which preventive behavior is assigned to individuals based on their demographics and “no predictors” case in which preventive behavior is assigned to individuals based on the average behavior observed in survey). The figure also compares cumulative infections across three vaccine efficacy levels (i.e., 20%, 40% and 60%) and two geographic regions (i.e., Montgomery, VA and Miami Dade, FL). Each box in the figure presents a five number summary: minimum, first quartile, median, third quartile, and maximum. Therefore, each box describes the distribution of cumulative infections produced by 25 replicates for each of the three experimental scenarios. Considering the median of 25 replicates to be the representative of a scenario, we observe that for a given vaccine efficacy, the scenarios with predictors (green boxes) consistently produce larger number of cumulative infections than those produced by scenarios without predictors (orange boxes). The red boxes show the base case where no intervention is applied. Therefore, every other scenario has a substantially lower number of cumulative infections as compared to the base case

Figure 5 provides a comparative evaluation of the cumulative infections observed in 25 simulation replicates, for each scenario, in both the regions. For all vaccine efficacy levels, and in both regions, the scenario with predictors performed worse than those without predictors. These findings imply that public policy will be misguided if inaccurate estimates of compliance to preventive behaviors are used. Assignment of behavioral interventions based on mean values will lead to more optimistic results about the epidemic, giving a false sense of security to public health decision makers.

### Age based infection rates

**Fig. 6:**
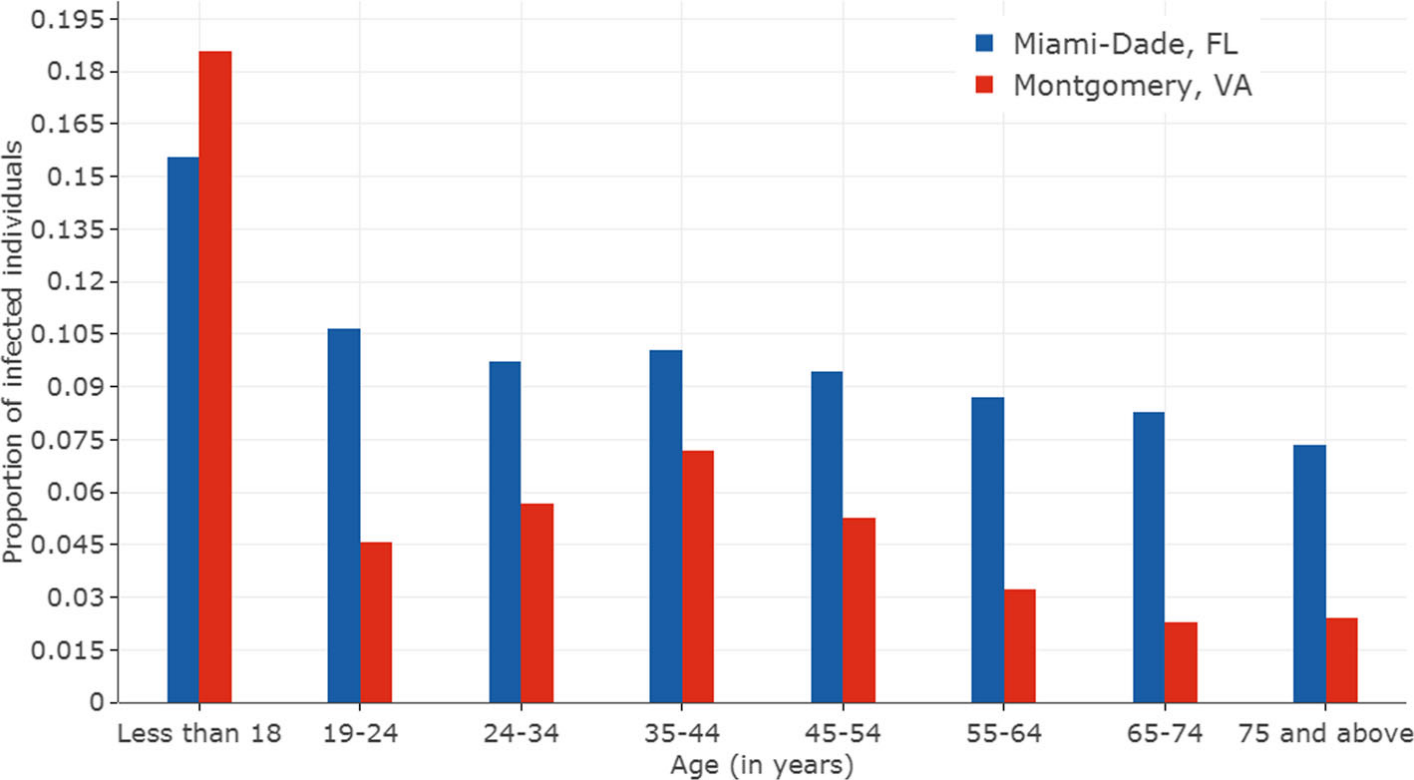
Influenza prevalence among different age groups for the two geographic regions, for the scenario “with predictors”. The vaccine efficacy is assumed to be 40%. Red bars correspond to Montgomery county, VA and Blue bars correspond to Miami Dade county, FL. The y-axis of the figures show the proportion of infected individuals in an age group. The x-axis of the figures show, 8 successive age groups (From ’Less than 18’ years to ’75 years and above’)

Figure 6 shows the prevalence of influenza (i.e. proportion infected in each age group) among different age groups in the populations for the two regions, for the scenario “with-predictors”. The vaccine efficacy is assumed to be 40%. For the youngest age group the prevalence of influenza is disproportionately high; and this effect is more pronounced in Montgomery than in Miami. Except for the 0–18 age group, in both Montgomery and Miami, the distribution of the infected population is well aligned with the overall population, higher for middle age groups and lower for older people. However in Montgomery, the age composition is quite different than Miami, i.e., a lot more young adults are present in the age group 19–24 and a lot less older individuals aged 65 and above.

We believe that the high incidence of infections in the youngest age group (0–18 years) is due to the following reasons: (1) People in the 0–18 age group have a higher number of contacts because they visit high-density locations such as school and daycare, and therefore a higher rate of exposure. Our earlier work has shown that children have a much higher network degree and social connectivity [36, 37]; (2) Children and young adults form a significant proportion of the total population in both the regions and interact more with other children and young adults. Age groups 0–24 make 30% of the population in Miami and 40% in Montgomery county.

Note that the compliance rate and the social network connectivity both play an important role in determining the epidemic outcome. Low compliance rates in the younger population, combined with a higher number of contacts result in disproportionately high levels of infections in the younger population. On the other hand, older people have a high level of compliance and a low level of connectivity, resulting in a proportionately lower number of infections. Note that this model does not consider the lower level of immunity and other co-morbidities among older people.

## Discussion

In the past, studies like [38] have tried to understand the impact of heterogeneity in parameters like susceptibility, infectivity and contact rates on the outbreak size, using ordinary differential equations. It has been shown that heterogeneous assignment of parameters, instead of a uniform assignment, affects the epidemic dynamics differently. In this study, we use an agent based model that explicitly models interactions among individuals in the true population, along with self-protective-behavior compliance rates that vary by demographics. The results show that uniform compliance versus demographics-based compliance lead to markedly different epidemic outcomes. These findings are consistent with the findings of the ODE modelling literature [38].

When compliance probabilities for protective behaviors are assigned based on average compliance, independent of individuals’ demographics, these behaviors are able to control the spread of the epidemic more effectively. However, if these behaviors are assigned based on the demographic characteristics, their effect on the epidemic outcomes is more subdued. This occurs even when the level of intervention is the same, i.e., on average, the same number of people are intervened. In other words, a more precise, demographic based assignment of compliance to behavioral interventions shows that the epidemic size and peak number of infections will be larger as compared to the case where mean values of compliance to protective behaviors observed in a sample are assigned to all individuals, independent of the demographic disparities that exist between them. The results hold for both the rural and urban regions in US.

The reason for this observation is that in the survey data, the compliance rates among the young adults are less than the average compliance rates. As evident from Fig. 2, young adults do not follow protective behaviors at the level reported by the mean compliance observed in the survey data. Given that these individuals have a much higher rates of mixing and contact time, lower compliance among them makes it easier to spread the infections. However this distinction is not captured when mean compliance is assigned to these cohorts. This makes the epidemic outcomes look better than the case when demographics based compliance rates are assigned.

This is a subtle but important distinction to understand from public health viewpoint because complex models are being increasingly used to inform public health policy [39]. A well characterized model for behavior adaptation guided by demographics will provide a more accurate prediction of the impact of preventative behaviors on epidemic outcomes. Infectious disease models are the result of a vast number of interacting social and biological processes in which complex models are necessary for accurate characterization of behaviors [40]. Our study addresses the need to more accurately parametrize models in which human behaviors are used to analyze the infectious disease dynamics. Such data-driven, analytic modeling also plays an important role in guiding future data collection, particularly by highlighting those data to which epidemic outcomes are most sensitive [11].

Lastly, there are many opportunities to extend this work. Here, we have used two geographic regions with significant differences in size, populations and their demographic distributions. We believe experimenting with other geographic regions could add more validity to this research and further highlight the role played by demographic disparities in the uptake of protective health behaviors. Similarly, in this study we have used a multinomial logit model for predicting individual’s health behavior adoption. Given the plethora of predictive modeling techniques available today, experimenting with other state of the art techniques for predicting compliance to health behaviors might be useful. Additionally, in this study, the initial survey does not capture individual attributes like psychographics and influence networks. These attributes might also play a role in determining an individual’s compliance decisions and can be explored in future research.

## Additional file

Additional file 1 Supplementary Information. Contains information regarding simulation compliance levels for different age groups and income groups in the two geographical regions. It also includes figures that describe the relationship between demographics and contact times. (PDF 228 kb)
