# Supplementary material for: Impact of demographic disparities in social distancing and vaccination on influenza epidemics in urban and rural regions of the United States
Source: BMC Infect Dis. 2019 Mar 4;19:221. doi: 10.1186/s12879-019-3703-2 (PMC6399923; doi:10.1186/s12879-019-3703-2)
Supplement: Supplementary file 1 — Supplementary Information. Contains information regarding simulation compliance levels for different age groups and income groups in the two geographical regions. It also includes figures that describe the relationship between demographics and contact times. (PDF 228 kb) [file 12879_2019_3703_MOESM1_ESM.pdf]

Supplementary Information: Impact of Demographic Disparities in Social Distancing and Vaccination on Influenza Epidemics in Urban and Rural Regions of the United States

Meghendra Singh[1], Prasenjit Sarkhel[2], Gloria J. Kang[1,3], Achla Marathe[7,8], Kevin Boyle[4], Pam Murray-Tuite[5], Kaja M. Abbas[6], and Samarth Swarup[7]

[1]Network Dynamics Simulation Science Laboratory, Biocomplexity Institute of Virginia Tech
[2]Department of Economics, University of Kalyani
[3]Department of Population Health Sciences, Virginia Tech
[4]Department of Agricultural and Applied Economics, Virginia Tech
[5]Department of Civil Engineering, Clemson University
[6]Department of Infectious Disease Epidemiology, London School of Hygiene & Tropical Medicine
[7]Biocomplexity Institute & Initiative, University of Virginia
[8]Department of Public Health Science, School of Medicine, University of Virginia

1 Supplementary Information

Table 1 shows the descriptive statistics about the demographic variables in the two model populations i.e., Montgomery County in Virginia and Miami-Dade County in Florida, used in this study. Figure 1 shows age and household income distributions of the two model populations. Figure 2 shows simulated compliance levels for different age groups in Montgomery county, VA and Miami Dade county in FL. Children in age group $0 - 18$ have been assigned the average compliance level of their family members. Since the survey only collects data for individuals who are older than 18 years, we assume that children behave the same as their respective family members and hence use the mean of the family members' compliance probabilities as a proxy.

Figure 3 shows the distribution of total contact duration among simulated individuals for different age and income groups in the two model populations.

Here, school-aged children between the ages of 5 and 17 years have contact durations considerably higher than other age groups for both the model populations. This is due to a large mixing of students at school. This also explains the high disease incidence rate among the "Less than 18" years age group. However that we do not observe a considerable difference in the contact duration for different household income groups.

Table 1: Descriptive statistics of demographics in the two model populations.

**Variable**	**Mean**	**Std. dev.**	**First Quartile**	**Median**	**Third Quartile**	**Max value**
Age (Montgomery, VA)	33.98	20.76	20	29	49	92
Age (Miami-Dade, FL)	37.43	22.36	19	37	54	94
Household Income (Montgomery, VA)	61.18K	55.75K	21.5K	49K	82.4K	649K
Household Income (Miami-Dade, FL)	69.34K	75.56K	26K	50K	86K	1500K
Household Size (Montgomery, VA)	2.37	1.22	1.0	2.0	3.0	13
Household Size (Miami-Dade, FL)	2.62	1.49	1.0	2.0	4.0	14
Number of children in a household (Montgomery, VA)	0.44	0.86	0.0	0.0	1.0	10
Number of children in a household (Miami-Dade, FL)	0.61	0.99	0.0	0.0	1.0	10

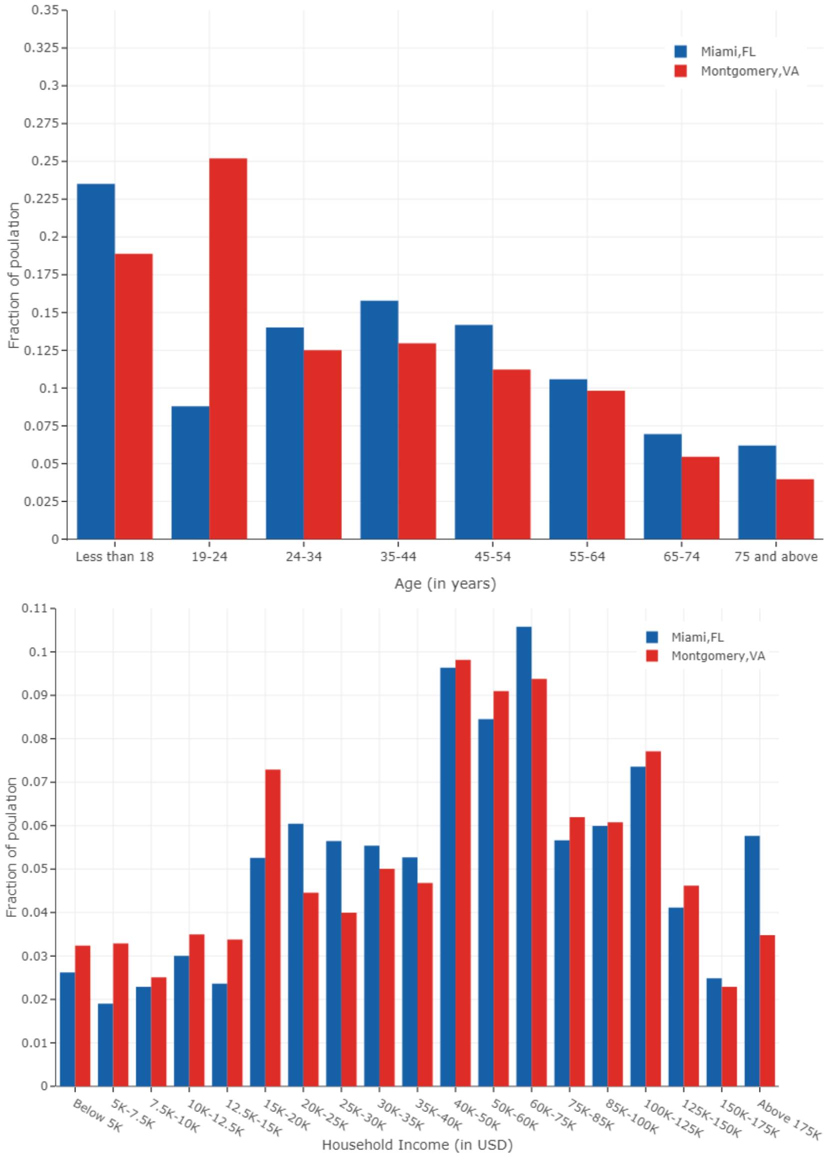

Figure 1: Age and household income distributions of the model populations for Montgomery County in Virginia and Miami-Dade County in Florida. Top plot shows the age distribution of the two populations and the bottom plot shows the household income distribution.

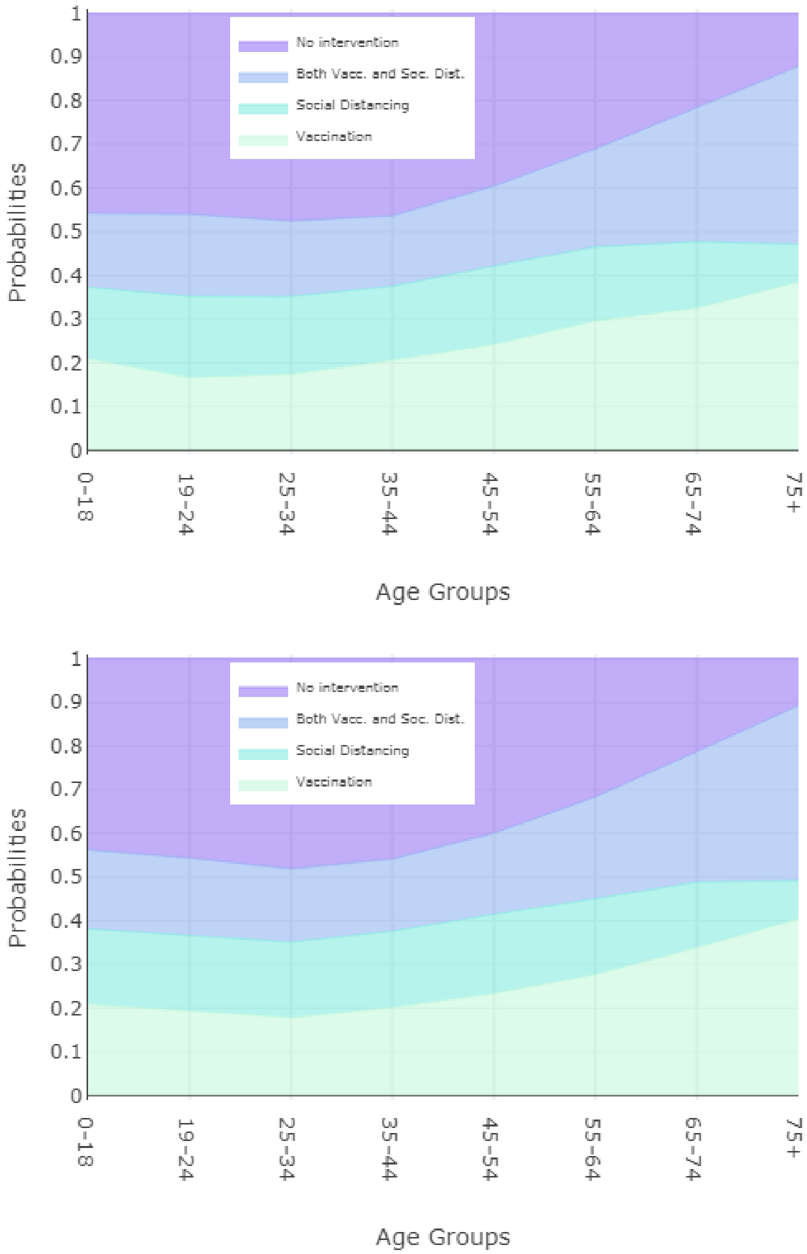

Figure 2: Simulated individuals' compliance to protective behaviors based on the multinomial logit model. Top plot shows age based compliance for Montgomery County in Virginia and the bottom plot shows it for the Miami-Dade County in Florida.

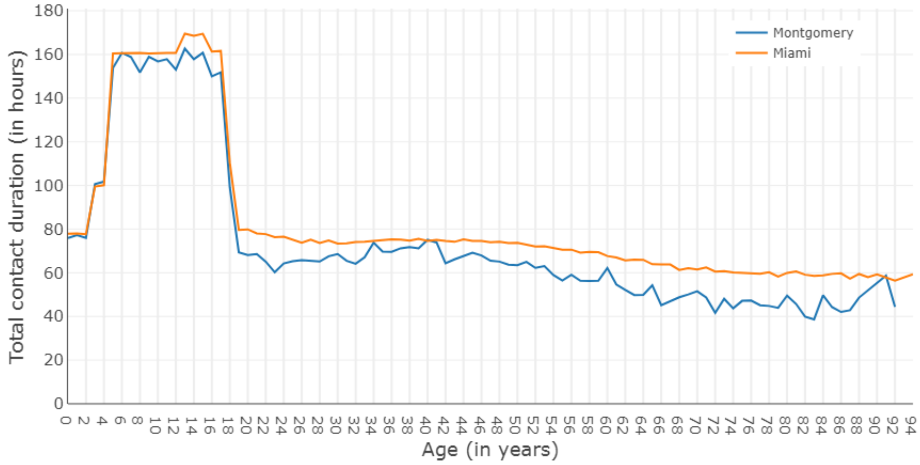

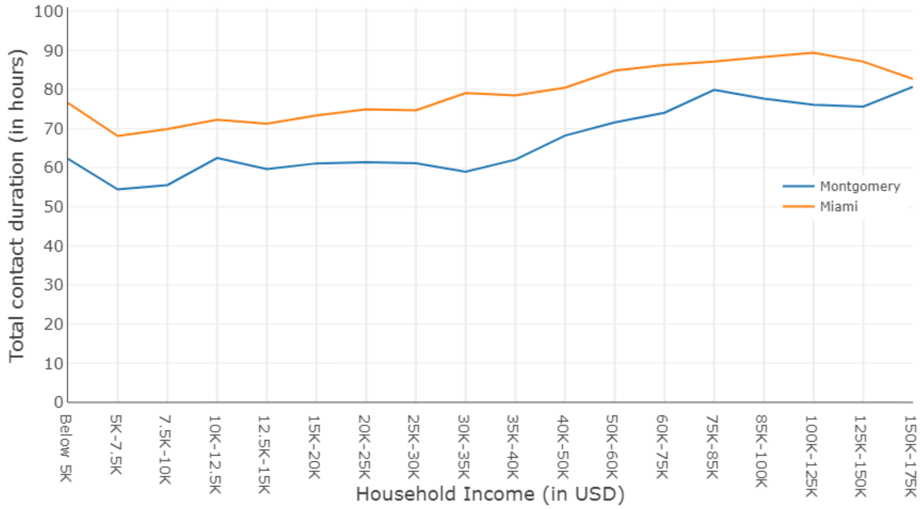

Figure 3: Total contact duration for different age and household income groups in Montgomery, VA and Miami-Dade, FL. Top plot shows age vesus total contact duration among simulated individuals. The vertical axis shows the mean of total contact duration for individuals having the age in years shown on the horizontal axis. Bottom plot shows household income versus total contact duration among simulated individuals. The vertical axis shows the mean of total contact duration for individuals having the household income in USD shown on the horizontal axis. Note that total contact duration for an individual is the sum of all contact times during a simulated day (i.e., during a day, if an individual spends t hours at a location with n other individuals, its total contact duration would be $t * n$ hours). Therefore, total contact time is usually more than 24 hours as it accounts for both the number of contacts and the duration of each contact.
